# Prevalence of Cataract Surgery and Visual Outcomes in Indian Immigrants in Singapore: The Singapore Indian Eye Study

**DOI:** 10.1371/journal.pone.0075584

**Published:** 2013-10-07

**Authors:** Preeti Gupta, Yingfeng Zheng, Tay Wan Ting, Ecosse L. Lamoureux, Ching-Yu Cheng, Tien-Yin Wong

**Affiliations:** 1 Singapore Eye Research Institute and Singapore National Eye Centre, Singapore, Singapore; 2 Centre for Eye Research Australia, University of Melbourne, Royal Victorian Eye and Ear Hospital, Victoria, Australia; 3 Department of Ophthalmology, Yong Loo Lin School of Medicine, National University of Singapore, Singapore, Spingapore; 4 Saw Swee Hock School of Public Health, National University of Singapore, Singapore, Singapore; 5 Centre for Quantitative Medicine, Office of Clinical Sciences, Duke-NUS Graduate Medical School, Singapore, Singapore; Duke University, United States of America

## Abstract

**Objective:**

To determine the prevalence of cataract surgery and factors associated with post-surgical visual outcomes in migrant Indians living in Singapore.

**Research Design and Methods:**

We conducted a population-based study in 3,400 Indian immigrants residing in Singapore−the Singapore Indian Eye Study (SINDI). All participants underwent comprehensive medical eye examination and a standardized interview. Post-operative visual impairment (VI) was defined as best-corrected or presenting visual acuity (BCVA or PVA) of 20/60 or worse.

**Results:**

The age- and gender-standardized prevalence of cataract surgery was 9.7% (95% confidence interval [CI]: 8.9%, 10.7%) in Singapore resident Indians. Post-operative VI defined by BCVA occurred in 10.9% eyes (87/795). The main causes of post-operative VI were diabetic retinopathy (20.7%), posterior capsular opacification (18.4%), and age-related macular degeneration (12.6%). Undercorrected refractive error doubled the prevalence of post-operative VI when PVA was used.

**Conclusions:**

The rate of cataract surgery is about 10% in Indian residents in Singapore. Socioeconomic variables and migration had no significant impact on the prevalence of cataract surgery. Diabetic retinopathy was a major cause of post-operative VI in migrant Indians living in Singapore. Uncorrected postoperative refractive error remains an efficient way to improve vision.

## Introduction

Cataract is the leading cause of visual impairment and blindness worldwide, accounting for more than 50% of the blindness cases [1], [2]. Driven by an increase in aged population and increasing demand for good vision, the number of persons who need cataract surgery is expected to rise worldwide. Cataract surgery is the most effective and widely used treatment for cataract and offers a cost-effective solution to this problem. Patients who undergo cataract surgery usually experience a significant improvement in visual functioning and quality of life [3]–[5]. However, many patients with cataract who live in middle and low income regions, especially in Asia, still have limited access to cataract surgery service. The major barriers to cataract surgery include cost [6], lack of knowledge about cataract [6]–[8], lack of transport and/or felt need [6], [8].

Globally, there is increased immigration between countries, with 200 million people who migrate from developing to developed countries every year [9]. The health of migrant population is a key public concern, as studies have shown that new immigrants living in developed countries have lower accessibility to health care and higher prevalence of chronic disease such as diabetes and hypertension [10]. India, the most populous country in South Asia, has seen a significant emigration wave each year, with increasing number living in the United States, UK and other countries [11]. It remains unclear whether migrant Indians have better accessibility to eye care service, such as cataract surgery, and have better visual outcomes after surgery, as compared with those still living in India.

Singapore is a newly developed country with three major ethnic groups (i.e., Chinese, Indians and Malays), with migrant Indians accounting for about 9.2% of the population [12]. The purpose of the present study was to determine the prevalence of cataract surgery and identify factors associated with post-operative visual outcome among migrant Indians living in Singapore, a major migration destination for Asians. Our data may provide new information in understanding the trends and impact of migration and health disparities attributable to cataract extraction in migrant populations.

## Materials and Methods

### Study Population and Design

We conducted a population-based study in migrant Indians residing in Singapore−the Singapore Indian Eye Study (SINDI). The SINDI examined 3,400 Indian adults aged 40–80 years living in Singapore between 2007 and 2009. Details of the study design, sampling plan, and methodology have been reported elsewhere [13]. In brief, the study was conducted in the southwestern part of Singapore, using the same study protocol as the Singapore Malay Eye Study [14]. On the basis of an age-stratified random sampling strategy, 6,350 names were selected. Of these, 4,497 individuals were determined to be eligible. A potential participant was considered “Ineligible” if the person moved from the residential address, had not lived there in the past 6 months, was deceased, or was terminally ill. Of the 4,497 eligible individuals, 3,400 participants took part in the study, representing a 75.6% participation rate. Of the non-participants, 1,021 (22.7% of eligible participant) declined to participate and 76 (1.7%) were not contactable. Non-participants on average were slightly older than participants (p<0.001), and there were no gender differences (p<0.28) between the 2 groups. Approval for the study was granted by the Singapore Eye Research Institute Institutional Review Board, and was conducted in accordance with the Declaration of Helsinki. Written informed consent was obtained from all participants before enrolment.

### Definition of Immigrant Status in SINDI

On the basis of the country of birth, participants were categorized as those born outside Singapore and were defined as ‘the first generation immigrants’. Those born in Singapore were defined as ‘the second or higher generation immigrants’. Among the 3,400 participants, 2,024 (59.5%) were born in Singapore, 812 (23.9%) were born in India (mostly South India, including Tamil Nadu, Kerala and Punjab), 496 (14.6%) were born in Malaysia and the remaining 68 (2.0%) were born in other south-east Asian countries like Pakistan, Bangladesh, Brunei and Sri Lanka; thus, 1,376 (40.4%) were classified as first generation immigrants and 2,024 (59.5%) were classified as second or higher generation immigrants.

### Study Procedures

An interviewer-administered questionnaire was used to collect information on socio-demographic, and lifestyle factors. All participants underwent an extensive and standardized examination procedure, which included visual acuity (VA) testing, a detailed clinical slit lamp and fundus examination before and after pupil dilation, and ocular imaging of the lens and retina.

Visual acuity (VA) was measured monocularly using a logarithm of the minimum angle of resolution (LogMAR) number chart (Lighthouse International, New York, USA) at a distance of 4 m. The presenting visual acuity (PVA) was ascertained with the participant wearing their “walk-in” optical correction (i.e. spectacles or contact lenses) if any, and best-corrected visual acuity (BCVA), in which refraction was corrected by trained and certified study optometrists, were obtained. If no numbers were read at 4 m, the participant was moved to 3, 2, or 1 m consecutively. If no numbers were identified on the LogMAR chart at all, VA was assessed as counting fingers, hand movements, perception of light, or no perception of light.

All participants underwent a detailed interview using standardized questionnaires. Information on birthplace, length of residence in Singapore, socioeconomic status (e.g. education, income, housing type), lifestyle risk factors (e.g. smoking), medication use and self-reported history of systemic disease were collected. Education categories were defined as 1) no formal education, 2) primary education included 1st to 5th grade, 3) secondary education included 6th to 8th grade, 4) polytechnics included 9th to 12th grade and university or college education. Participants were asked if a health provider had ever told them that they have cataract. Those who responded “yes” were classified as having “known cataract”. Clinical assessment of lens status and the presence of aphakia or pseudophakia were determined with slit-lamp examination. Patients with any cataract surgery were defined as the ones with lens extraction in either or both eyes. The presence or absence, and the clarity, of the posterior lens capsule, were also determined in pseudophakic eyes. If the study participants required any treatment for postoperative VI, they were referred to the nearest eye care facility with a referral letter.

### Definition and causes of Poor Visual Outcomes

Post-operative visual impairment (VI) was defined by a presenting visual acuity (PVA) of 20/60 (6/18) or less, or a best-corrected visual acuity (BCVA) of 20/60 (6/18) or less, in the operated eye either in unilateral cataract extraction or in bilateral extractions. Primary causes of VI were assessed by the study ophthalmologist on the basis of information obtained from clinical history and examination, and if necessary, from ocular imaging data (lens and retina) [14]. If there was more than one cause, the most treatable or preventable cause was selected as the principal cause for the person. For example, if there was PCO in 1 eye and optic neuropathy in the fellow eye, the principal cause was PCO for that person. Under-corrected refractive error in the operated eye was defined when there was an improvement of at least 0.2 log MAR (2 lines equivalent) in the best-corrected VA in comparison with the presenting VA. Glaucoma was diagnosed and classified using the International Society of Geographic and Epidemiological Ophthalmology (ISGEO) scheme [15], based on findings from gonioscopy, optic disc characteristics, and visual fields results. Age-related macular degeneration was graded from retinal photographs according to the Wisconsin grading system [16]. Diabetic retinopathy was graded from retinal photographs using a modification of Arlie House classification system for the Early Treatment Diabetic Retinopathy Study (ETDRS) [17]. Amblyopia was diagnosed in eyes with visual impairment if no obvious structural or pathological causes can be detected by physical examination.

### Statistical Analysis

All statistical analyses were performed using the Statistical Package for the Social Science (V. 17.0, SPSS Inc., Chicago, IL). A p value <0.05 indicated statistical significance. Prevalence estimates and 95% confidence intervals (CIs) of cataract surgery were calculated and standardized to the Singaporean Indians using the 2010 Singapore Census data (http://www.singstat.gov.sg). The prevalence and causes of post-operative VI were determined for all the operated eyes. Data from both eyes were analyzed together, using the Generalized Estimating Equation (GEE) model which accounts for the correlation between the right and left eyes, to assess the factors associated with post-operative VI. For our analysis, we only fitted the basic model with each of the listed socioeconomic factors, adjusting for age and gender. As each of them is an independent model, without including each other (e.g. other socioeconomic factors), collinearity is thus not an issue.

## Results

Of the 3,400 participants in SINDI, 12 subjects had neither slit lamp examination nor lens data, and were therefore excluded, leaving 3,388 participants for analysis. There were 486 subjects (14.3%) with cataract extraction in at least one eye (795 eyes in total, including 10 eyes with aphakia and 785 eyes with pseudophakia) and 309 subjects (9.1%) with bilateral extractions (7 eyes with aphakia and 611 eyes with pseudophakia). There were 3 subjects who had aphakia in one eye and pseudophakia in the other eye and were counted in both groups. Among first-generation immigrants, the average duration of residence in Singapore was 41.9 years (standard deviation [SD] = 17.2). Their father’s birthplaces mainly included Tamil Nadu (46.5%), Kerala (9.2%) and Punjab (7.7%), and Malaysia (11.5%), and their mother’s had similar birthplace distributions. The rate of cataract surgery was not associated with length of residence (OR = 1.01, 95% confidence interval [CI] 0.92%, 1.10%). Compared to first-generation immigrants, second-generation immigrants were generally younger, were more likely to be smokers and had better socio-economic factors such as higher education, income and better housing facilities (Table S1). **Table 1** demonstrates the age and gender adjusted prevalence of cataract surgery among first and second or higher generation Indian immigrants living in Singapore. The prevalence of cataract surgery among the immigrants was significantly associated with older age.

**Table 1:** Prevalence of cataract surgery by country of birth: 1^st^ and 2^nd^ or higher generation Indian immigrants living in Singapore.

	All persons		1^st^ generation Immigrants		2^nd^ or higher generation Immigrants	
Surgery	N	No. (%) ofcataract surgery	N	No. (%) ofcataract surgery	N	No. (%) ofcataract surgery
Any cataract surgery*						
40–49 years	893	4 (0.4)	262	0 (0.0)	631	4 (0.6)
50–59 years	1094	49 (4.5)	304	13 (4.3)	790	36 (4.6)
60–69 years	890	166 (18.7)	432	88 (20.4)	457	78 (17.1)
70+ years	511	267 (52.3)	373	201 (53.9)	138	66 (47.8)
*P*-value for trend		<0.001		<0.001		<0.001
Total	3388	486 (14.3)	1371	302 (22.0)	2016	184 (9.1)
Adjusted prevalence (95% CI)		9.7 (8.9, 10.7)†		9.9 (8.7, 11.4)†		9.1 (7.8, 10.7)†
Bilateral cataract surgery§						
40–49 years	893	2 (0.2)	262	0 (0.0)	631	2 (0.3)
50–59 years	1094	19 (1.7)	304	5 (1.6)	790	14 (1.8)
60–69 years	890	93 (10.4)	432	52 (12.0)	457	41 (9.0)
70+ years	511	195 (38.2)	373	148 (39.7)	138	47 (34.1)
*P-*value for trend		<0.001		<0.001		<0.001
Total	3388	309 (9.1)	1371	205 (15.0)	2016	104 (5.2)
Adjusted prevalence (95% CI)		6.1 (5.5, 6.9)†		6.4 (5.5, 7.6)†		5.5 (4.5, 6.8)†

N = number of individuals in the age group; CI = confidence interval.

* Any cataract surgery was defined as lens extraction (pseudophakia or aphakia) in either or both eyes.

† Age- and gender-adjusted to the Indian adult population from the 2010 Singapore census.

§ Bilateral cataract surgery was defined as lens extraction (pseudophakia or aphakia) in both eyes.

**Table 2** shows the factors associated with cataract surgery between the first and second or higher generation Indian migrants living in Singapore. In first generation, age, female gender and diabetes (all p<0.05) were found to be significantly related with a higher odds of having undergone cataract surgery. However those with polytechnic or university education were less likely to undergo cataract surgery. In second generation, age, diabetes and hypertension are the factors significantly associated with higher rate of cataract surgery.

**Table 2:** Socioeconomic and systemic factors associated with cataract surgery in 1^st^ generation and 2^nd^ or higher generation Indian immigrants living in Singapore.

	Presence of any cataract surgery among1^st^ generation immigrants		Presence of any cataract surgery among2^nd^ or higher generation immigrants	
Factor	N (%)	Age-gender adjustedOR (95% CI)	N (%)	Age-gender adjustedOR (95% CI)
Age, per 10 years	1343 (22.5)	5.21 (4.23, 6.41)*	1983 (9.3)	4.82 (3.94, 5.90)*
Gender (Female)	644 (24.7)	1.56 (1.14, 2.13)*	1006 (8.9)	0.96 (0.68, 1.35)
Living alone (Yes)	75 (28.0)	0.86 (0.46, 1.59)	91 (9.9)	0.61 (0.28, 1.35)
Education				
No education	187 (46.5)	1.00	124 (21.8)	1.00
Primary education	554 (26.7)	0.70 (0.47, 1.06)	989 (10.2)	1.33 (0.74, 2.39)
Secondary education	244 (16.4)	0.66 (0.39, 1.12)	560 (6.6)	1.06 (0.55, 2.06)
Polytechnics	126 (7.9)	0.38 (0.16, 0.88)*	226 (5.8)	1.08 (0.47, 2.46)
University	230 (7.0)	0.48 (0.24, 0.94)*	82 (4.9)	0.79 (0.24, 2.64)
Monthly income				
Less than S$1000	563 (35.9)	1.00	502 (18.5)	1.00
S$1000 to S$2000	163 (20.9)	0.71 (0.44, 1.15)	361 (7.8)	0.74 (0.45, 1.20)
More than S$2000	371 (12.4)	0.77 (0.50, 1.17)	814 (5.9)	0.83 (0.54, 1.27)
Housing type				
1–2 room HDB	91 (38.5)	1.00	66 (15.2)	1.00
3–4 room HDB	754 (22.5)	1.18 (0.69, 2.01)	1218 (9.4)	1.43 (0.63, 3.24)
5-room/executive HDB or private housing	496 (19.6)	0.96 (0.55, 1.69)	696 (8.5)	1.40 (0.60, 3.26)
Language of interview				
Tamil	486 (31.9)	1.00	378 (15.6)	1.00
English	765 (15.2)	0.86 (0.62, 1.21)	1483 (7.3)	1.04 (0.68, 1.59)
Malay	83 (34.9)	1.06 (0.60, 1.89)	122 (13.9)	0.94 (0.48, 1.81)
Diabetes (Yes)	479 (32.6)	1.52 (1.11, 2.08)*	626 (16.3)	2.06 (1.45, 2.93)*
Hypertension (Yes)	825 (30.1)	1.34 (0.92, 1.97)	1058 (13.7)	1.58 (1.05, 2.37)*
Reading ability (Yes)	1196 (20.2)	0.77 (0.49, 1.21)	1846 (8.5)	0.82 (0.46, 1.46)
Writing ability (Yes)	1172 (19.8)	0.89 (0.59, 1.35)	1833 (8.5)	1.05 (0.60, 1.84)
Length of residence, per decade	1343 (22.5)	1.05 (0.92, 1.20)	1983 (9.3)	1.05 (0.63, 1.74)

OR = odds ratio; CI = confidence interval; HDB = Housing Development Board, *p<0.05.

**Table 3** presents the socioeconomic and systemic factors associated with post-operative VI, defined as PVA ≤20/60, by immigrant status. Except education, no significant factors were found to be associated with post-operative VI among both the groups.

**Table 3:** Socioeconomic and systemic factors associated with post-operative visual impairment in the 1^st^ and 2^nd^ or higher generation Indian migrants living in Singapore.

	Postoperative VI among 1^st^ generationimmigrants (n = 139 eyes)	Postoperative VI among 2^nd^ generationimmigrants (n = 64 eyes)
Factor	Age-gender adjusted OR (95% CI)	Age-gender adjusted OR (95% CI)
Age, per 10 years	1.00 (0.69, 1.46)	1.03 (0.74, 1.44)
Gender (Female vs Male)	1.32 (0.84, 2.06)	1.18 (0.63, 2.21)
Education		
No education	1.00	1.00
Primary education	0.47 (0.28, 0.77)*	0.58 (0.23, 1.47)
Secondary education	0.70 (0.33, 1.47)	0.29 (0.08, 1.02)
Polytechnics	0.57 (0.17, 1.96)	0.53 (0.11, 2.59)
University	0.29 (0.09, 0.89)*	4.53 (0.66, 31.3)
Monthly Income		
Less than S$1000	1.00	1.00
S$1000 to S$2000	0.53 (0.23, 1.18)	1.21 (0.50, 2.91)
More than S$2000	1.12 (0.58, 2.16)	0.70 (0.28, 1.74)
Housing type		
1–2 room HDB	1.00	1.00
3–4 room HDB	0.87 (0.45, 1.67)	1.54 (0.29, 8.23)
5-room/executive HDB or private housing	0.73 (0.35, 1.51)	1.36 (0.25, 7.49)
Diabetes (Yes vs No)	1.10 (0.70, 1.72)	1.79 (0.94, 3.38)
Hypertension (Yes vs No)	0.81 (0.46, 1.43)	0.800 (0.35, 1.84)
BMI (kg/m^2^)	0.94 (0.88, 1.00)	0.99 (0.92, 1.07)
Length of residence, per decade	1.11 (0.90, 1.36)	0.69 (0.23, 2.05)

VI = visual impairment, defined as presenting visual acuity ≤20/60. OR = odds ratio; CI = confidence interval.

* p<0.05.

**Table 4** summarizes the main causes of post-operative VI as defined by PVA and BCVA in both groups. Among Singaporean Indians, post-operative VI was found in 203 eyes (25.5%) of the operated eyes as defined by PVA, and in 87 eyes (10.9%) as defined by BCVA. Under-corrected refractive error accounted for more than half (57.1%) of post-operative VI as defined by PVA. The primary causes of post-operative VI defined by BCVA were diabetic retinopathy (20.7%), posterior capsular opacification (18.4%) and age related macular degeneration (12.6%). There was no significant difference (p = 0.417) between the generations in the primary causes of post-operative VI.

**Table 4:** Causes of post-operative visual impairment in the 1^st^ and 2^nd^ or higher generation Indian migrants living in Singapore.

	Postoperative visual impairment					
Causes	PVA≤20/60All persons(n = 203 eyes)	BCVA≤20/60All persons(n = 87 eyes)	PVA ≤20/601^st^ generation(n = 139)	PVA≤20/602^nd^ generation(n = 64)	BCVA≤20/601^st^generation(n = 56)	BCVA ≤20/602^nd^generation(n = 31)
Uncorrected refractive error	116 (57.1)	0 (0.0)	83 (59.7)	33 (51.6)	0 (0.0)	0 (0.0)
Diabetic retinopathy	18 (8.9)	18 (20.7)	12 (8.6)	6 (9.4)	12 (21.4)	6 (19.4)
Posterior capsular opacification	16 (7.9)	16 (18.4)	12 (8.6)	4 (6.3)	12 (21.4)	4 (12.9)
Age-related macular degeneration	11 (5.4)	11 (12.6)	9 (6.5)	2 (3.1)	9 (16.1)	2 (6.5)
Corneal disease	8 (3.9)	8 (9.2)	5 (3.6)	3 (4.7)	5 (8.9)	3 (9.7)
Glaucoma	9 (4.4)	6 (6.9)	4 (2.9)	2 (3.1)	4 (7.1)	2 (6.5)
Non-glaucomatous optic neuropathy	7 (3.4)	7 (8.0)	4 (2.9)	3 (4.7)	4 (7.1)	3 (9.7)
Others	7 (3.4)	7 (8.0)	4 (2.9)	3 (4.7)	4 (7.1)	3 (9.7)
Macular disease	6 (3.0)	6 (6.9)	3 (2.2)	3 (4.7)	3 (5.4)	3 (9.7)
Amblyopia	6 (3.0)	4 (4.6)	2 (1.4)	2 (3.1)	2 (3.6)	2 (6.5)
Other retinal disease	4 (2.0)	4 (4.5)	1 (0.7)	3 (4.7)	1 (1.8)	3 (9.7)

PVA = presenting visual acuity; BCVA = best-corrected visual acuity.

“Other” included one individual with pterygium, one with phthisis, one with trauma and one with myopic maculopathy. The exact cause in three individuals cannot be determined.

## Discussion

We reported the prevalence of cataract surgery and factors associated with post-operative visual outcomes in a population-based study among the first and second generation Indian immigrants living in Singapore. The age-standardized prevalence of any cataract extraction was 9.7% (9.9% and 9.1% among first and second generation immigrants, respectively). Older persons and those with diabetes were more likely to have cataract surgery, but cataract surgery was not associated with most of the socioeconomic factors and the generation of immigrants. Diabetic retinopathy was the most common primary cause of post-operative VI (defined by BCVA). Furthermore, one in four eyes (203/795) with cataract surgery continued to have VI, of which more than 50% (116/203) is largely due to the lack of full refractive correction.

The age-standardized rate of cataract surgery in Indians living in Singapore (9.7%) was twice as higher as those in rural Central India (4.7%) [18], similar to those reported in urban India (10.5%) [19] but lower than those reported in a study done in Navsari in Gujarat in 2009 [20] and recent Andhra Pradesh Study in 2012 [21] which showed a prevalence of 17.6% and 15.4% respectively (**Table 5**).

**Table 5:** Comparison of prevalence, risk factors and outcomes of cataract surgery from selected population-based studies in Asia.

			Cataract Surgery			Post-operative Visual Impairment	
Study	N	Age, yrs	Crude Prevalence, %	Adjusted Prevalence,* %	Risk factors associated	%, basedon BCVA	Causes
**Urban Asians**							
Chennai Urban [19]	3850	>40	10.5	–	–	15.6	Refractive error, PCO, CME
APEDS [27]	2522	≥50	14.6	–	–	–	Refractive error, surgical
The Liwan Eye Study [33]	1405	>50	4.4	–	–	23.3	Retinal abnormalities, glaucoma, uncorrected aphakia or refractive error, PCO
Beijing Eye Study [34]	4378	≥40	2.86	–	Age, angle-closure glaucoma,hemorrhagic retinopathy	10.5	Refractive error, hemorrhagic retinopathy, PCO
Hong Kong Study [3]	3441	≥60	9.0	–	–	–	Refractive error, AMD, glaucoma, PCO
Tanjong Pagar Survey [23]	1232	40–81	11.1	5.5	Diabetes	–	–
SiMES [22]	3280	40–80	8.7	5.0	Older age, male sex, diabetes	10.8	Refractive error, DR, glaucoma
SINDI (current study)	3400	40–80	14.3	9.7	Older age, diabetes	10.9	Refractive error, DR, PCO, AMD
**Rural Asians**							
Navsari Gujarat Study [20]	4738	≥50	17.6	–	Older age, literacy, urban residence	25.5	Refractive error, PCO, macular degeneration
Andhra Pradesh Study [21]	7281	≥50	15.4	–	Older age, availing free surgery	19.3	Refractive error, surgical complications, posterior segment disorders
Chennai Rural [19]	3924	>40	13.5	–	–	27.7	Refractive error, PCO, CME
ACES [28]	5411	≥50	11.8	11.3	Literacy, male sex	16.9	Refractive error, AMD, surgical complications
CIEMS [18]	4711	>30	6.4	4.7	Age, diabetes, female sex, shorteraxial length	36	Refractive error, surgical complications, PCO
The China Nine- Province Survey [35]	45747	>50	2.09	–	Older age, female gender, lack ofeducation, province	36.2	PCO, refractive error, retinal disorders

APEDS = Andhra Pradesh Eye Disease Study; SiMES = Singapore Malay Eye Study; SINDI = Singapore Indian Eye Study; ACES = Aravind Comprehensive Eye Study; CIEMS = Central India Eye & Medical Study; BCVA = best corrected visual acuity; PCO = posterior capsular opacification; CME = cystoid macular edema; AMD = age-related macular degeneration; DR = diabetic retinopathy.

“−”: not reported.

* Age-standardized to the Indian adult population from the 2010 Singapore Census.

Interestingly, the age-standardized rates of cataract surgery in Indians are twice as high as those from ethnic Malays (5.0%) [22] and Chinese (5.5%) [23] residing in Singapore. Similarly, a study using Medisave (government-administered medical savings fund) database between 1991 and 1996 found that cataract extraction rates in Singapore were highest for Indians (396.5 per 100000/year), compared to Chinese (371.2 per 100000/year) and Malays (237.2 per 1000000/year) [24]. The underlying reasons for such an ethnic difference are not clear and may be multiple. First, the level of knowledge and awareness of cataract surgery care may vary among these three ethnic groups. Second, Indians in Singapore have a higher prevalence of diabetes (21.6%) [25], an established risk factor for cataract [26], compared to the other two ethnic groups (Malays, 17.1%, Chinese, 11.5%) [25], which may explain the rate difference among ethnic groups in part. Furthermore, prevalence of age-related cataract and cataract surgery rates seems to be much higher in persons living on the Indian subcontinent [20], [21], and studies have reported much higher cataract surgery rates in Indian immigrants to the UK compared to the Caucasian population irrespective of their diabetes status. Therefore, individuals of Indian ancestry may be genetically more susceptible to cataract, compared to other ethnic groups. Further studies would be needed clarify whether genetic factors contribute to the observed rate difference.

Regarding risk factors for cataract surgery, older age and the presence of diabetes were significantly associated with cataract surgery in both the generations, consistent with other population-based studies [19], [23], [27]. A higher prevalence was also associated with female gender in first generation and with presence of hypertension in second generation immigrants. This is consistent with evidence from a previous study in rural India which reported associations of female gender and arterial hypertension along with age and diabetes with higher cataract surgery rate [18]. In contrast, except higher levels of education (polytechnic and university) in first generation immigrants, none of the socioeconomic variables had any influence on the prevalence of cataract surgery among both the generations. It appears that educated persons are more skeptical to any surgery as their major concerns are the outcomes and risks of surgery. The lack of socioeconomic gradient in cataract surgery is also seen in previous studies in Indians living in rural and urban India [28]. This appears to suggest that cost is not the major barrier to cataract surgery and highlights the need to identify other key determinants of cataract surgery in this ethnic group.

Despite significant differences in characteristics of our first and second generation immigrants, their socio-economic status: education, income, housing type and average duration of residence in Singapore (Table S1), our study showed that immigrant generations had no influence on the prevalence, risk factors and post-operative visual outcomes of cataract surgery among the two generations of migrant Indians. However, one could expect no differences in the prevalence and outcomes of cataract surgery between the two generations as life style and behavior factors may not largely change with a generation after immigration. In fact, to study migrant disparity it would have been more logical to study differences between first and third (or higher) generation Indians and see if the surgery rate increases, and factors are different for those who are several generations after immigration. However, Singapore was established less than 50 years and thus there are insufficient numbers in third (or higher) generation.

One fourth of the post-operated had VI based on PVA, and the most common cause was under corrected refractive error (57.1%). The magnitude of under-corrected refractive error in our study was higher than that reported in CIEMS (41.8%) [18] but lower than that reported in SiMES (60%) [22]. In our study, 10.9% of all the pseudophakic eyes had post-operative VI. Similar results were also revealed in various other studies in Asian countries [27], [28]. Postoperative monitoring (by simple refraction) to ensure good visual acuity outcome is necessary to eliminate VI among the already operated individuals.

We reported that 10.9% of operated eyes (87/795) had best-corrected VI and ocular conditions such as diabetic retinopathy, posterior capsular opacification and age related macular degeneration were the leading causes **(** **Table 4** **)**. Previous studies in Singapore found higher prevalence of diabetes [25], [29] and diabetic retinopathy [30] in migrant Asian Indians compared with that of the other two ethnic groups (Malays and Chinese), suggesting the importance of environmental factors that accompany migrant as well as possible genetic susceptibility. Thus, the fact that diabetic retinopathy and posterior capsular opacification were the primary causes of almost 40% of visual impairment in the cataract-operated eyes in this study cohort is of significant concern. This is in contrast to studies from the western world, where age-related macular degeneration is found to be the leading cause of visual impairment after cataract surgery [31], [32]. The public health impact of the increasing prevalence of diabetes and diabetic retinopathy on cataract surgery services demand and outcomes, as shown by our data, will be relevant for planning healthcare strategies in Singapore and many other Asian countries.

Strengths of this study include its population-based large sample, high participation rate (75.6% response) and detailed classification of the first-and the second generation immigrants. Nevertheless, our study findings are subject to a number of limitations. First, due to our cross-sectional study design, we were unable to determine the causal relationships between the various risk factors and postoperative visual outcomes. Second, the possibility of selection bias, although unlikely, could not be totally excluded in our cohort. However, according to the results of 2010 Singapore census, our study sample is a fair representation of the Singapore population in terms of age distribution, housing type and socioeconomic status and there was no significant differences in sampling locations between the respondent and non-respondent group [10]. Third, our study may not be fully comparable to the other Indian studies in India, given the differences in study designs, population characteristics, sampling frame and data collection, and also the differences in the health care systems between Singapore and India. Lastly, we did not assess the type of cataract surgery performed on our subjects and also had no information on when it was performed.

In conclusion, the age-standardized prevalence of cataract surgery among Singaporean Indians is 9.7%. Socioeconomic measures and migration had no significant impact on the prevalence of cataract surgery among the first and second generation Indian immigrants in Singapore. Under-corrected refractive error, diabetic retinopathy and posterior sub capsular opacification are among the leading causes of post-operative VI among Singaporean Indians. Thus proper post-operative refractions, adequate follow-up and provisions of glasses will greatly improve cataract surgical outcome in Singapore.

## Supporting Information

Table S1 **Characteristics of the first- and second-generation Indian immigrants living in Singapore.**
(DOCX)
