# Supplementary material for: Prevalence of Cataract Surgery and Visual Outcomes in Indian Immigrants in Singapore: The Singapore Indian Eye Study
Source: PLoS One. 2013 Oct 7;8(10):e75584. doi: 10.1371/journal.pone.0075584 (PMC3792142; doi:10.1371/journal.pone.0075584)
Supplement: Table S1 — Characteristics of the first- and second-generation Indian immigrants living in Singapore. (DOCX) [file pone.0075584.s001.docx]

**Table S1.** Characteristics of the first- and second-generation Indian immigrants living in Singapore.

|  | 1^st^ generation (N=1371) | 2^nd^ generation (N=2016) | P value* |
| --- | --- | --- | --- |
| Age (per year) | 61.6 (10.9) | 55.1 (8.5) | <0.001 |
| Female gender | 659 (48.1) | 1030 (51.1) | 0.084 |
| Living alone (Yes) | 77 (5.6) | 91 (4.5) | 0.147 |
| Average duration of residence in Singapore (years) | 41.9 (17.2) | 54.7 (8.9) | <0.001 |
| BMI (per kg/m^2^) | 25.7 (4.4) | 26.5 (4.9) | <0.001 |
| HbA1c (%) | 6.4 (1.2) | 6.5 (1.5) | 0.075 |
| SBP (per mmHg) | 137.5 (19.9) | 134.0 (19.1) | <0.001 |
| DBP (per mmHg) | 76.5 (9.9) | 78.0 (10.2) | <0.001 |
| Total cholesterol (per mmol/l) | 5.0 (1.1) | 5.3 (1.1) | <0.001 |
| HDL cholesterol (per mmol/l) | 1.1 (0.3) | 1.1 (0.3) | 0.075 |
| LDL cholesterol (per mmol/l) | 3.2 (0.9) | 3.4 (1.0) | <0.001 |
| Current smoker (yes) | 145 (10.6) | 350 (17.4) | <0.001 |
| Diabetes (Yes) | 491 (36.9) | 633 (32.3) | 0.006 |
| Hypertension (Yes) | 842 (61.5) | 1077 (53.5) | <0.001 |
| Reading ability (Yes) | 1224 (89.3) | 1877 (93.1) | <0.001 |
| Writing ability (Yes) | 1199 (87.5) | 1864 (92.5) | <0.001 |
| Education  No education  Primary education  Secondary education  Polytechnics  University | 190 (13.9)  566 (41.3)  248 (18.1)  130 (9.5)  235 (17.2) | 126 (6.3)  1008 (50.0)  570 (28.3)  226 (11.2)  84 (4.2) | <0.001 |
| Monthly income  Less than S$1000  S$1000 to S$2000  More than S$2000 | 783 (58.7)  171 (12.8)  380 (28.5) | 775 (39.4)  365 (18.6)  826 (42.0) | <0.001 |
| Housing type  1-2 room HDB  3-4 room HDB  5-room/executive HDB or private housing | 92 (6.7)  771 (56.3)  506 (37.0) | 67 (3.3)  1241 (61.6)  705 (35.0) | <0.001 |

BMI=Body mass index; SBP = systolic blood pressure; HbA1C = hemoglobin A1C; HDL = high-density lipoprotein; LDL = low-density lipoprotein; SGD=Singapore dollar.

Data presented are means (standard deviations) or number (%), as appropriate for variable.

*P value, comparing the differences between the 1st and 2nd generation immigrants, based on chi-square test or t test, as appropriate.
